# Anti-atherogenic Modification of Serum Lipoprotein Function in Patients with Rheumatoid Arthritis after Tocilizumab Treatment, a Pilot Study

**DOI:** 10.3390/jcm9072157

**Published:** 2020-07-08

**Authors:** Daniela Greco, Roberta Gualtierotti, Pasquale Agosti, Maria Pia Adorni, Francesca Ingegnoli, Matteo Rota, Franco Bernini, Pier Luigi Meroni, Nicoletta Ronda

**Affiliations:** 1 Department of Food and Drug, University of Parma, 43124 Parma, Italy; danielagreco@hotmail.it (D.G.); mariapia.adorni@unipr.it (M.P.A.); f.bernini@unipr.it (F.B.); nicoletta.ronda@unipr.it (N.R.); 2 Department of Pathophysiology and Transplantation, Università degli Studi di Milano and Fondazione Luigi Villa, 20122 Milan, Italy; roberta.gualtierotti@unimi.it (R.G.); pasquale.agosti@unimi.it (P.A.); 3 Fondazione IRCCS Ca’ Granda Ospedale Maggiore Policlinico, Angelo Bianchi Bonomi Hemophilia and Thrombosis Center, 20122 Milan, Italy; 4 UOC Reumatologia Clinica, ASST Pini-CTO, Department of Clinical and Community Sciences, Research Center for Adult and Pediatric Rheumatic Diseases, Università degli Studi di Milano, 20122 Milano, Italy; francesca.ingegnoli@unimi.it; 5 Department of Molecular and Translational Medicine, University of Brescia, 25123 Brescia, Italy; matteo.rota@unibs.it; 6 Immunorheumatology Research Laboratory, IRCCS Istituto Auxologico Italiano, 20145 Milano, Italy

**Keywords:** cardiovascular disease, tocilizumab, rheumatoid arthritis, atherosclerosis, lipoproteins, cellular cholesterol, macrophages, cholesterol efflux, cholesterol loading

## Abstract

Lipid metabolism derangement contributes to increased cardiovascular risk in Rheumatoid Arthritis (RA). It is still debated whether and how tocilizumab, an interleukin-6 receptor inhibitor used in active RA, impacts cardiovascular risk. We studied the effect of tocilizumab on the regulation of macrophage cholesterol homeostasis, measuring patient serum ability to respectively load (cholesterol loading capacity, CLC) and discharge (cholesterol efflux capacity, CEC) cells with cholesterol. Patients with RA (*n* = 8) were studied before and after 4 and 12 weeks of tocilizumab treatment. CLC was measured by a fluorimetric assay of intracellular cholesterol content in human macrophages and CEC was measured for the three main pathways, mediated by the transporters Scavenger Receptor class B-type I (SR-BI), ATP binding cassette-G1 (ABCG1) and -A1 (ABCA1) in specific cell models. After 12 weeks of tocilizumab treatment, serum LDL cholesterol levels were increased, while CLC was reduced. HDL cholesterol levels were unchanged, but CEC was significantly ameliorated for the SR-BI and ABCG1 pathways with respect to baseline. Tocilizumab reduces LDL pro-atherogenic potential despite increasing their serum levels and increases HDL protective activity in RA. The data of our pilot study suggest that tocilizumab regulates lipoprotein function in selected patient populations and lay the groundwork for future larger studies.

## 1. Introduction

Rheumatoid arthritis (RA) is associated with accelerated atherosclerosis and increased cardiovascular risk, due to various mechanisms including inflammation, autoimmune reactions, and lipid metabolism derangement, all impacting arterial wall homeostasis. Interleukin (IL)-6, one of the key pro-inflammatory cytokines involved in RA pathogenesis, is independently associated with cardiovascular risk in the general population [1]. Tocilizumab is a monoclonal antibody directed against IL-6 receptor used in active RA, but concerns have been raised about its cardiovascular safety, because its use is associated with the increase in circulating LDL levels [2]. However, growing evidence supports the view that tocilizumab does not increase cardiovascular risk [3,4]. Our hypothesis is that functional lipoprotein changes occurring during tocilizumab treatment may offset or even divest LDL increase of its negative significance and/or associate to an amelioration of HDL anti-atherogenic properties in patients with RA. A key step in atherosclerosis is foam cell formation, resulting from the unbalance between macrophage cholesterol loading and efflux. Cholesterol loading capacity (CLC) is serum ability, mostly due to LDL, to deliver cholesterol to cells. Native serum LDL interact mainly with LDL receptors, while modified LDL are taken up by cells through various other receptors, such as scavenger receptors, which are not downregulated by intracellular cholesterol content increase. Cholesterol efflux capacity (CEC) is serum ability, mainly due to HDL, to promote cell cholesterol efflux. CEC is considered a key marker of HDL function and a measure of reverse cholesterol transport [5] that has been inversely associated with the incidence and prevalence of cardiovascular disease [6,7]. Macrophage-cholesterol efflux is mostly mediated by the transporters Scavenger Receptor class B-type I (SR-BI), ATP binding cassette-G1 (ABCG1), and -A1 (ABCA1); each of these transporters deliver cholesterol preferentially to specific HDL subfractions. As shown in animal models and clinical studies [8], CEC has a preventive role on atherosclerosis progression and cardiovascular events, regardless of HDL levels [6,7,8,9,10]. Modifications of lipoprotein composition and function contributing to the increased cardiovascular risk in RA have been described [11,12,13]. In RA, in fact, LDL and HDL oxidation, the activity of certain autoantibodies, altered pattern of proteins associated to HDL are all reported mechanisms leading to lipoprotein pro-atherogenicity though the increase in intracellular macrophage cholesterol. Thus, tocilizumab treatment, through its anti-inflammatory activity, might improve qualitative characteristics of circulating lipoproteins and help restore their function. The aim of this pilot study was to explore the effect of tocilizumab treatment on serum CLC and serum CEC specifically mediated by the SRBI, ABCG1, and ABCA1 transporters, to verify our hypothesis in a small group of patients in order to set the rationale and sample size for larger studies.

## 2. Materials and Methods

### 2.1. Patients

We studied eight consecutive adult patients with established RA, attending the outpatient clinic of Gaetano Pini Institute in Milan, Italy, and fulfilling the 2010 American College of Rheumatology (ACR) criteria for RA [14], starting intravenous tocilizumab treatment at a dose of 8 mg/kg every 4 weeks. Six patients were taking corticosteroids, two were on metothrexate, one on leflunomide and one on hydroxychloroquine. Demographic and clinical characteristics of the study population are reported in Supplementary Material, Table S1. Written informed consent was obtained from all subjects and the study was approved by the Ethics Committee of Fondazione IRCCS Ca’ Granda Ospedale Maggiore Policlinico, Milan, Italy on 01/25/2013 (Studio Numero 1751-ID 55800). The study conformed with the Helsinki Declaration of 1964, as revised in 2013, concerning human and animal rights. Patients or the public were not involved in the design, or conduct, or reporting or dissemination plans of our research.

### 2.2. Disease Activity

At baseline and after four weeks of intravenous tocilizumab therapy, disease activity was measured based on the evaluation of 28 joints and erythrocyte sedimentation rate (DAS28) [15]. We did not report on the CDAI because at the time of the study this parameter was not yet included in our current clinical practice.

### 2.3. Laboratory Measurements

Blood samples were collected at baseline and after 4 and 12 weeks from starting tocilizumab, immediately before the due infusion. Plasma levels of C-reactive protein (CRP), erythro-sedimentation rate (ESR), the circulating concentration of total cholesterol, HDL-cholesterol (HDL-C), LDL-cholesterol (LDL-C), and triglycerides were measured at baseline and after 12 weeks. Serum CLC and CEC were measured at baseline, after 4 and after 12 weeks from the beginning of treatment.

### 2.4. Cholesterol Loading Capacity (CLC) Measurement

For serum CLC measurement, THP-1 cells were cultured in 12-well plates in the presence of 50 ng/ml Phorbol 12-Myristate 13-Acetate (PMA) (Sigma Aldrich, Milano, Italy) for 72 h to allow differentiation into macrophages. Cells were then exposed for 24 h to 5% patient serum. Cell cholesterol content was measured by fluorimetric detection in cell lysates [16]. DNA content in the cell lysates was measured through the deoxyribose-diphenylamine reaction method [17]. CLC was defined as macrophage cholesterol content in the cell lysates after exposure of cells to serum and expressed as mg/μg DNA.

### 2.5. Cholesterol Efflux Capacity (CEC) Measurement

For SR-BI CEC, cholesterol efflux was measured using rat hepatoma Fu5AH in the absence or presence of a specific SR-BI inhibitor (Block Lipid Transfer-1 10 μM, purchased from ChemBridge, San Diego, CA, USA). For ABCG1 CEC, ABCA1-mediated efflux was measured using J774 macrophages incubated with or without cAMP (Sigma Aldrich, Milano, Italy), which stimulates ABCA1 expression. For ABCG1 CEC, cholesterol efflux was evaluated in Chinese hamster ovary cells transfected or not with the human abcg1 gene for ABCG1-mediated efflux. In all assays, the cells were labelled with [1,2-3H]-cholesterol (PerkinElmer, Milano, Italy) for 24 h, incubated with 0.2% bovine serum albumin in medium for 24 h and treated for 4 or 6 h with 1% or 2% (*v*/*v*) whole serum, depending on the cholesterol efflux pathway to be analyzed. Serum CEC results were expressed as a percentage of total radioactivity incorporated by the cells that was released in the supernatant. A pool of human normolipidaemic sera was tested in each assay as reference standard 1 and its CEC was used to normalize the patient samples values from the different experiments, in order to correct for the inter-assay variability. A second pool of human normolipidaemic sera as reference standard 2 was tested in each assay and its CEC, after normalization, was the index of the intra-assay variability (13).

### 2.6. Statistical Analyses

Statistical analyses were performed using GraphPad Prism version 5.0. Each experiment was performed in triplicate. Variables were expressed as median and interquartile range (IQR). Specific pathway-mediated CEC and CLC values were compared between time points with the Wilcoxon signed-rank test for paired samples. The estimate of the median difference and its 95% confidence interval were calculated with SPSS 26 using the Hodges-Lehmann method. The effect size for the comparison of CLC and specific pathway-mediated CEC values at 12 weeks of treatment with tocilizumab as compared with baseline values was computed as a quantitative measure of variability using the estimated median difference divided by its standard deviation. We also evaluated the number of patients required to detect an effect size of 0.3, considered to be small, using a two-sided non-parametric Wilcoxon signed-rank test for paired data assuming a uniform distribution.

## 3. Results

### 3.1. Clinical and Laboratory Parameters

At baseline, median DAS28 (IQR) was 3.5 (2.9–4.3), CRP plasma levels were 5.3 μg/mL (2–9.3), and ESR was 51 mm in the first hour (27–57.8). After 4 weeks, median DAS28 (IQR) was 1.5 (1.2–2), CRP 0.5 μg/mL (0.3–1.8), and ESR 1 mm in the first hour (0–6.3). Lipid profile before and after tocilizumab treatment is reported in Supplementary Material Table S2. The only significant variation was the increase of median LDL-C mg/dL (IQR) from 132 (125–146) at baseline to 161 (144–179) (*p* = 0.028) after 12 weeks of treatment.

### 3.2. Serum CLC

**Figure 1:**
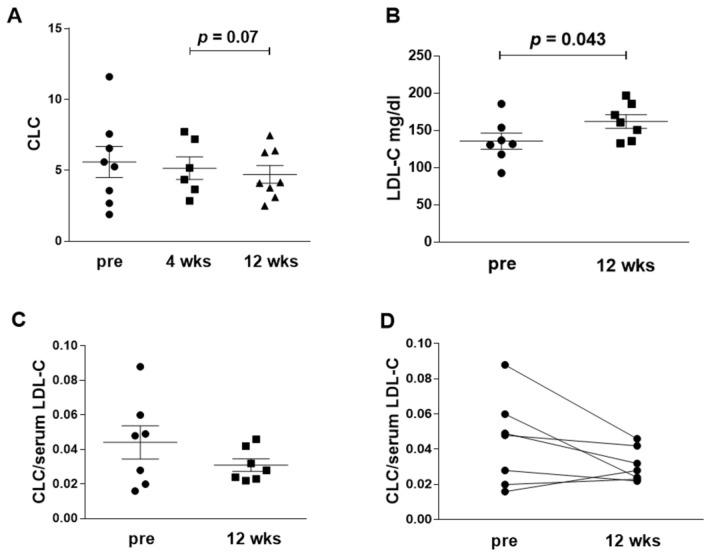
Serum cholesterol loading capacity (CLC) before and after tocilizumab treatment. (**A**): CLC, expressed as mg cholesterol/μg DNA in macrophage cells extracts, at baseline (pre), after 4 weeks (4 wks) and 12 weeks (12 wks) of tocilizumab treatment (**B**): LDL-cholesterol (LDL-C) serum level (mg/dL) at baseline and after 12 weeks of treatment. (**C**): the panel reports the ratio CLC/LDL-C in each patient at the two time points. (**D**): the panel shows the change with time of CLC/LDL-C ratio in each patient. Statistical significance was calculated with the Wilcoxon test for paired samples with skewed distribution.

Tocilizumab treatment, despite the increase in LDL-C levels after 12 weeks, was not associated with an increase in serum CLC (Table 1 and Figure 1, panels A and B).

Conversely, a not statistically significant decrease was observed in CLC. The lack of statistical significance with respect to the baseline might be due, not only to the small number of patients, but also to the wide distribution of CLC values before treatment. Normalizing CLC for serum LDL-C levels the decrease was more evident: CLC/LDL-C was 0.05 (0.02–0.06) at baseline and 0.03 (0.02–0.04) after 12 weeks (Figure 1C). Analysing in detail the modifications of CLC for each patient upon treatment, it was noted that the normalized CLC decrease was greater in patients with higher baseline values (Figure 1D).

### 3.3. Serum CEC

**Figure 2:**
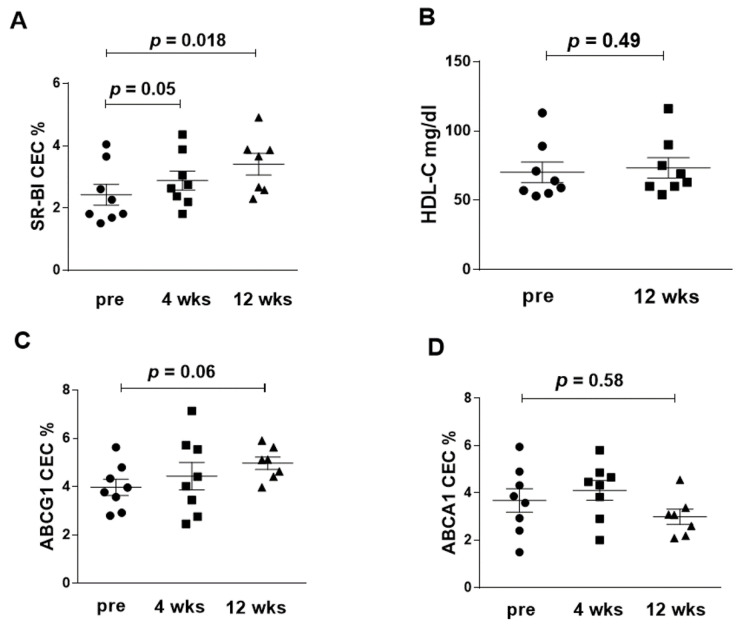
Serum cholesterol efflux capacity (CEC) before and after tocilizumab treatment. (**A**): CEC, expressed as percent effluxed of total cell cholesterol, specifically mediated by the SR-BI transporter. (**B**): HDL-C serum levels (mg/dL) at baseline and after 12 weeks of treatment. (**C**): CEC, expressed as percent effluxed of total cell cholesterol, specifically mediated by the ABCG1 transporter (**D**): CEC, expressed as percent effluxed of total cell cholesterol, specifically mediated by the ABCA1 transporter. Statistical significance was calculated with Wilcoxon test for paired samples with skewed distribution.

SR-BI-mediated CEC was increased after tocilizumab, both at 4 (*p* = 0.05) and 12 weeks (*p* = 0.018) of treatment (Figure 2A), despite total HDL-C serum levels being unmodified (Figure 2B).

ABCG1-mediated CEC showed an increase, close to statistical significance, after 12 weeks (*p* = 0.06) (Figure 2C). At the same time point, ABCA1-mediated CEC displayed a non-significant tendency to a reduction compared to the baseline (Figure 2D).

### 3.4. Sample Size Estimate for a Larger Study

Using the two-sided non-parametric Wilcoxon signed-rank test for paired data assuming a uniform distribution, we computed that about 100 patients would ensure detecting for all parameters a small effect size of 0.3 with a study power equal to 80% and a type I error of 5, including a dropout percentage of 20% or a 90% study power with no dropouts.

## 4. Discussion

The prevention and therapy of cardiovascular disease is complex and challenging. An important emerging approach is the one focusing on patient population specificities, to optimize the treatment both in terms of efficacy, safety, and sustainability.

Patients with autoimmune diseases are at high cardiovascular risk and present with specific lipid abnormalities, both in terms of circulating levels and lipoprotein quality and function, which deserve a focused approach.

For example, in RA patients, LDL levels alone do not reflect cardiovascular risk. In fact, RA disease activity associates with accelerated atherosclerosis despite the low LDL levels compared to age and sex-matched healthy subjects [18], resulting from the inflammation-related LDL hypercatabolism [19]. Moreover, as a result of the LDL alterations occurring during systemic inflammation and/or in the presence of autoantibodies, LDL proatherogenicity might be increased [11,20,21]. Similarly, the HDL dysfunction largely described in systemic inflammatory or autoimmune diseases, including RA, leads to a decrease in their atheroprotective properties [11,12,13].

Tocilizumab, an interleukin-6 receptor inhibitor, increases circulating LDL levels [2] without increasing the risk of major cardiovascular events in RA patients [3,4]. Tocilizumab would blunt the LDL hypercatabolic state and reduce the expression of LDL receptor on hepatocytes via a proprotein convertase subtilisin/kexin type-9 (PCSK9)-mediated mechanism [22,23,24]. It also improves endothelial function, reduces prothrombotic state [25] and oxidative stress, modulates adipokines, attenuates monocyte prothrombotic and inflammatory phenotype, and induces abridged NETosis [26].

Our data suggest that the improvement of lipoprotein function could be a possible adjunctive mechanism of cardiovascular protection following tocilizumab treatment. Regarding LDL function, we found that CLC did not increase together with serum LDL levels after treatment. Moreover, it decreased particularly in subjects with the highest baseline values. This might reflect a reduction of inflammation-dependent LDL proatherogenicity following tocilizumab treatment. Indeed, recent clinical studies suggest that tocilizumab reduces LDL-associated proatherogenic factors, such as lipoprotein-associated phospholipase A2, lipoprotein (a), and cholesteryl ester transfer protein [26,27,28]. Thus, in this specific population of RA patients treated with tocilizumab or possibly other anti-rheumatic drugs, as we reported for an anti-TNF agent [16], LDL increase should not be automatically interpreted as proatherogenic, due to the favorable changes in LDL function. Regarding HDL function, the improvement of SR-BI and ABCG1-mediated serum CEC that we observed after treatment, again independent of HDL-C circulating levels, likely reflects the improvement of their antiatherogenic properties, consistent with a previous report on the amelioration of HDL CEC measured as total cholesterol efflux after tocilizumab treatment [24]. As SR-BI and ABCG1 interact preferentially with mature HDL, whereas ABCA1 with poor cholesterol, immature HDL, our findings suggest that tocilizumab treatment is associated with a restored HDL maturation. Indeed, systemic inflammation has been reported to associate with reduced HDL maturation [29]. Interestingly, in a previous work on RA patients, we have shown that ABCG1-mediated CEC inversely correlates with DAS28 [13]. Overall, as HDL particles interacting with the cholesterol transporters SR-BI and ABCG1 constitute the large majority of circulating HDL, their function impacts significantly on macrophage cholesterol metabolism. As for LDL, tocilizumab might modulate HDL composition, reducing HDL binding to inflammatory proteins in RA patients (e.g., serum amyloid A4 and complement C4) [30].

Our study has some limitations: the small number of subjects, partly due to the monocentric design of the study, and short observation time. The small number of patients in this study limited the potential for detailed statistical analyses, but allowed to show statistically significant differences in lipoprotein function before and after tocilizumab treatment. Therefore, this pilot study provides a useful direction for further confirmatory studies in larger groups of subjects, that we computed to be about 100 patients. We report on CLC and on three specific CEC pathways, never explored before in this experimental setting, providing a clue for mechanistic studies of the links between the control of inflammation and lipid metabolism. Of note, patient blood samples were collected at the trough levels, before drug administration, thus excluding a hypothetical direct action of tocilizumab on the cells used in our assays.

Overall, the important qualitative beneficial changes in lipoproteins after tocilizumab treatment may contribute to explain the cardiovascular safety of tocilizumab in RA and correctly interpret LDL levels modifications in terms of cardiovascular risk. Further prospective cohort studies with longer follow-up and a larger sample size will be useful to better understand all the effects of tocilizumab on lipid metabolism in RA. Moreover, these original data and approach, although referring to a small sample of patients, could be of interest in view of both clinical and research activity on the prevention and treatment of cardiovascular disease in patients with systemic inflammatory conditions.

## Figures and Tables

**Table 1:** Serum cholesterol loading capacity (CLC) and cholesterol efflux capacity (CEC) measured by three different pathways at baseline and after 4 and 12 weeks of treatment with tocilizumab.

	Baseline	After 4 Weeks	After 12 Weeks	Median Difference Baseline vs. 12 Weeks [95% CI]
**CLC** **(IQR)**	5.4 [2.9, 7.1]	4.7 [3.4, 7.3]	4.1 [3.3, 6.4] ^#^	−0.95 [−2.61, 0.4]
**SR-BI** **(IQR)**	2.0 [1.7, 3.4]	2.7 [2.2, 3.7] *	3.7 [2.6, 3.9] **	0.87 [0.48, 1.62]
**ABCG1 (IQR)**	3.9 [3.1, 4.7]	4.2 [2.9, 5.7]	5.1 [4.4, 5.6]	0.92 [−0.05, 2.32]
**ABCA1 (IQR)**	4.0 [2.7, 5.1]	4.7 [3.4, 5.2]	3.3 [2.8, 3.6]	−0.34 [−1.33, 0.97]

CLC = cholesterol loading capacity; SRBI Scavenger Receptor class B type I-mediated CEC; ABCG1 = ATP binding cassette G1-mediated CEC; ABCA1= ATP binding cassette A1-mediated CEC; CI confidence interval; # *p* = 0.075 vs. 4 weeks * *p* = 0.05 vs. baseline; ** *p* = 0.018 vs. baseline.

## Supplementary Materials

The following are available online at https://www.mdpi.com/2077-0383/9/7/2157/s1. Table S1: Baseline demographic and clinical characteristics of patients with established rheumatoid arthritis starting tocilizumab, Table S2: Lipid profile of patients with established rheumatoid arthritis before and after 12 weeks from starting tocilizumab treatment.
