# Supplementary material for: Anti-atherogenic Modification of Serum Lipoprotein Function in Patients with Rheumatoid Arthritis after Tocilizumab Treatment, a Pilot Study
Source: J Clin Med. 2020 Jul 8;9(7):2157. doi: 10.3390/jcm9072157 (PMC7408911; doi:10.3390/jcm9072157)
Supplement: Supplementary file 1 [file jcm-09-02157-s001.pdf]

Table S1: Baseline demographic and clinical characteristics of patients with established rheumatoid arthritis starting tocilizumab.

	Patients (n=8)
Female % (n)	88 (7)
Median age in years [IQR]	61 [55-65]
Median disease duration in years [IQR]	15.5 [14-24.8]
Median DAS28 [IQR]	3.5 [2.9-4.3]
Smoking habit % (n)	38 (3)
RF positivity % (n)	88 (7)
ACPA positivity % (n)	75 (6)
Median number of previous biologic drugs [IQR]	2.5 [2-4]
Median weeks of wash-out before starting tocilizumab [IQR]	7 [5-17]
Patients treated with daily equivalent prednisone dose ≥5 mg/day % (n)	75 (6)
Median prednisone daily dose [IQR]	5 [4.4-7.5]

ACPA anti-citrullinated peptide antibodies; DAS28 disease activity 28 joints; IQR interquartile range; RF rheumatoid factors

Table S2: Lipid profile of patients with established rheumatoid arthritis before and after 12 weeks from starting tocilizumab treatment.

	Baseline	After 12 weeks
Median total cholesterol mg/dL [IQR]	220 [206-239]	231 [228-252]
Median HDL mg/dL [IQR]	62 [57-76]	66 [60-79]
Median LDL mg/dL [IQR]	132 [125-146]	161 [144-179]*
Median TGL mg/dL [IQR]	128 [90-178]	151 [93-186]

TGL triglycerides; HDL high density lipoprotein; IQR interquartile range; LDL low density lipoprotein; *p=0.028 vs baseline.
